# Nadir Oxygen Delivery During Pediatric Cardiopulmonary Bypass and Postoperative Acute Kidney Injury: A Pilot Cohort Study

**DOI:** 10.3390/children13070893

**Published:** 2026-07-03

**Authors:** Demet Kangel, Burcu Çevlik, İncila Ali, Ezgi Direnç Yücel, Eymen Recep, Tarık Demir, Ali Can Hatemi, Erkut Öztürk

**Affiliations:** 1 Department of Pediatric Cardiology, University of Health Sciences, Basaksehir Cam and Sakura City Hospital, Istanbul 34480, Turkey; drburcudrl@gmail.com (B.Ç.); erkut.ozturk@sbu.edu.tr (E.Ö.); 2 Department of Anaesthesiology and Reanimation, University of Health Sciences, Basaksehir Cam and Sakura City Hospital, Istanbul 34480, Turkey; incilali@hotmail.com (İ.A.); ezgidirenckulunk@yahoo.com (E.D.Y.); 3 Department of Pediatric Cardiovascular Surgery, University of Health Sciences, Basaksehir Cam and Sakura City Hospital, Istanbul 34480, Turkey; dreymenrecep@gmail.com (E.R.); tarikdemir@uskudar.edu.tr (T.D.); alican.hatemi@sbu.edu.tr (A.C.H.)

**Keywords:** pediatric, congenital heart surgery, acute kidney injury, oxygen delivery

## Abstract

**Background:** Among infants undergoing cardiac surgery, AKI may affect as many as 40% of this population and carries an unfavorable prognosis. A range of contributors has been implicated, including early age, cyanotic physiology, and extended time on cardiopulmonary bypass (CPB). Nadir-indexed oxygen delivery (DO_2_) is an important determinant for early detection of hypoperfusion and anaerobic metabolism during CPB. This study aimed to explore the relationship between nadir DO_2_ during pediatric CPB and postoperative acute kidney injury. **Methods:** Between 1 October 2024 and 1 December 2024, we enrolled 40 children who underwent cardiac surgery with CPB [median age 6 months (IQR 4–8), median weight 5.5 kg (IQR 5–7 kg)] into an observational cohort. DO_2_ was tracked intraoperatively for every patient, and pre- as well as intraoperative variables were examined for independent links to AKI. Postoperative outcomes were then compared between patients who did and did not develop AKI. **Results:** In our patient population (*n* = 40), 16 patients (40%) developed AKI according to the pRIFLE criteria (75% risk; 18.7% injury; 6.2% failure; no patient was in the loss or end-stage renal disease categories). Lower nadir DO_2_ values were associated with postoperative AKI in this exploratory pilot cohort. ROC analysis identified an exploratory nadir DO_2_ threshold of 360 mL/min/m^2^ (sensitivity 70%, specificity 80%) associated with postoperative AKI. Because this threshold was both derived and evaluated within the same cohort without validation, it should be regarded only as a preliminary, hypothesis-generating observation with no implication of clinical applicability and requires validation in larger independent cohorts. **Conclusions:** In this exploratory pilot cohort, lower nadir DO_2_ during cardiopulmonary bypass was independently associated with postoperative AKI. An exploratory ROC-derived threshold of 360 mL/min/m^2^ was identified; however, this threshold should be considered hypothesis-generating rather than clinically validated. The consistency of findings across both dichotomous and continuous DO_2_ analyses strengthens the robustness of the observed association, although larger prospective studies are required to externally validate this threshold, which at present carries no implication of clinical applicability.

## 1. Introduction

Renal dysfunction frequently complicates the postoperative course of children recovering from congenital cardiac procedures and is regularly seen in the intensive care setting. Published cohorts place its frequency anywhere between roughly one-third and two-thirds of patients, and the burden falls disproportionately on neonates in the days after surgery [1,2].

Recognized risk factors in this setting include prematurity, younger age, high-dose inotrope requirements, longer CPB duration, the depth of hypothermia, surgical complexity, and postoperative low cardiac output syndrome [3,4].

DO_2_ reflects how much oxygen reaches peripheral tissues and is a product of circulatory flow and the oxygen-carrying capacity of blood. Once it drops past a critical point, aerobic energy generation can no longer be sustained and the tissues shift toward anaerobic metabolism, so holding DO_2_ above that point throughout bypass is essential [5,6].

The literature increasingly suggests that low renal blood flow and inadequate oxygen delivery during pediatric CPB are important modifiable predictors of postoperative AKI. Several studies have demonstrated an association between low-indexed oxygen delivery and cardiac surgery-associated AKI in infants and children; however, uncertainties remain regarding optimal DO_2_ thresholds across different pediatric subgroups, temperature strategies, and surgical complexities [5,7,8,9]. Therefore, the present study was designed as an exploratory, hypothesis-generating pilot investigation to evaluate nadir (lowest) DO_2_ as a predictor of AKI during pediatric CPB.

## 2. Materials and Methods

This retrospective study included infants with congenital heart disease (CHD) who underwent heart surgery with CPB in our unit between 1 October 2024 and 1 December 2024. Enrollment began in October 2024, coinciding with the institutional adoption of a standardized intraoperative oxygenation protocol during cardiopulmonary bypass, targeting a relatively constant arterial PaO_2_ of approximately 250 mmHg. This approach was intended to ensure a more homogeneous perfusion strategy across all included patients. No changes in surgical technique, perfusion equipment, monitoring devices, or data-recording practices occurred during the study period. Inclusion criteria were as follows: (1) age < 1 year, (2) use of cardioplegia and aortic cross-clamp (ACC), and (3) admission to the pediatric cardiac intensive care unit (PCICU) following heart surgery. Exclusion criteria included (1) patients who underwent circulatory arrest and regional perfusion with additional cannulation, (2) patients lacking data necessary to calculate DO_2_, and (3) patients with a history of renal disease or who underwent preoperative peritoneal dialysis or hemodialysis. All clinical and perfusion data were generated during routine patient care between October and December 2024. The retrospective analysis of these previously collected, anonymized clinical data was subsequently approved by the institutional ethics committee (Approval No: 231; Approval Date: 13 August 2025), in accordance with institutional and national ethical requirements and the principles of the Declaration of Helsinki. Because the approval covered the retrospective use of routinely collected clinical data, the requirement for informed consent was waived. During the study period, all consecutive infants undergoing congenital heart surgery requiring cardiopulmonary bypass were screened for eligibility. Patients meeting the predefined inclusion criteria and without exclusion criteria were included in the final analysis. No matching procedures were performed. Because of the retrospective design, no patients were lost to follow-up for the primary endpoint assessment.

- For each patient, the following variables were extracted from the hospital information system and entered into a standardized case report form:
- Preoperative body weight, sex, diagnosis of cardiac disease (cyanotic and acyanotic, single ventricle and biventricular), and tests that included serum creatinine (Cr) and blood urea nitrogen (BUN) values.
- Operative data (cardiopulmonary bypass time, aortic cross-clamp time, blood gases, lactate, lowest DO_2_, average DO_2_) and surgical complexity score (RACHS-1).
- Postoperative data, ICU follow-up data (serum creatinine, blood urea nitrogen, lactate, the duration of mechanical ventilation, mortality, morbidity, vasoactive inotrope score (VIS), and classification system data (pRIFLE) for the presence of AKI.

In perioperative anesthesia management, drug usage followed the protocol used in our clinic. According to this protocol, in children under six months, 0.1 mg/kg midazolam, 10 micrograms/kg/fentanyl, and 0.6 mg/kg rocuronium were used for induction; during maintenance, remifentanil was given at 0.2 micrograms/kg/min, rocuronium at 5 micrograms/kg/min, and sevoflurane was administered at 1–1.2 minimum alveolar concentration. In children over six months, 0.1 mg/kg midazolam, 10 micrograms/kg/fentanyl, and 0.6 mg/kg rocuronium were used for induction; during maintenance, remifentanil was given at 0.25 micrograms/kg/min, rocuronium at 5 micrograms/kg/min, sevoflurane at 1–1.2 minimum alveolar concentration, and dexmedetomidine at 0.5 micrograms/kg/hr. The effects of neuromuscular agents were antagonized with sugammadex. Remifentanil and rocuronium were discontinued after sternum closure. Sevoflurane was stopped before skin closure. Dexmedetomidine was continued until transfer to the pediatric cardiac intensive care unit [10].

The arterial line was placed in the ascending aorta, whereas venous drainage was secured either bicavally or through the right atrial appendage according to the operation performed. A single-use hollow-fiber membrane oxygenator (Affinity Pixie; Medtronic, Minneapolis, MN, USA) together with a roller pump (Stockert-5; Sorin Group, Munich, Germany) made up the circuit.

Myocardial protection relied on Del Nido solution, delivered cold at a 1:4 dilution; the induction dose was 20 mL/kg, followed by 10 mL/kg every 60 min. A 20 mg furosemide dose preceded the start of CPB in each case. Target roller-pump flow was set at 180 mL/kg/min for neonates, 150 mL/kg/min for infants weighing under 10 kg, and 2.4 L/min/m^2^ for those at or above 10 kg. Conventional ultrafiltration ran during bypass, with modified ultrafiltration added once the patient was weaned.

Throughout the procedure, hemoglobin (g/dL), hematocrit (%), arterial oxygen tension (PaO_2_; mmHg), and arterial oxygen saturation (SaO_2_) were obtained from a blood gas analyzer (ABL800; Radiometer Co., Copenhagen, Denmark). Core temperature (nasopharyngeal, bladder, or rectal), pump flow (L/min), and invasive mean arterial pressure were documented every 10 min. Missing values were present in isolated perfusion measurements from two patients and were replaced using the nearest subsequent available measurement to maintain continuity of the DO_2_ calculations. DO_2_ was calculated at each recorded measurement time point (approximately every 10 min during CPB) using the following equation:

DO_2_ (mL/min/m^2^) = indexed pump flow (L/min/m^2^) × [1.36 × Hb (g/L) × SaO_2_ + 0.031 × PaO_2_ (mmHg)]. Arterial oxygen saturation (SaO_2_) was entered as a decimal fraction (e.g., 99% = 0.99) rather than as a percentage. Hemoglobin was recorded in g/dL and multiplied by 10 to convert to g/L for use in the equation. The nadir DO_2_ was defined as the lowest calculated value, and the average DO_2_ was defined as the mean of all calculated values obtained during CPB [11].

The Risk Adjustment in Congenital Heart Surgery (RACHS-1) scores were used to classify the procedures. The RACHS-1 classification ranges from category 1 to category 6 [12].

The inotropes used and their doses were recorded, and the Vasoactive Inotrope Score (VIS) of the patients was calculated daily. VIS was calculated by the following formula: VIS = Dopamine dose (μg/kg/min) + Dobutamine dose (μg/kg/min) + 100 × Epinephrine dose (μg/kg/min) + 10 × Milrinone dose (μg/kg/min) + 10,000 × Vasopressin dose (μg/kg/min) + 100 × Norepinephrine dose (μg/kg/min) [13].

The pRIFLE system was used to define AKI. The pRIFLE criteria are based on postoperative fall in glomerular filtration rate (GFR) compared to baseline GFR. GFR was calculated using the modified Schwartz formula as follows: eGFR (mL/min/1.73 m^2^) = k × Ht/SCr, (k: 0.413, Ht: height (cm), SCr: serum creatinine (mg/dL)) [14].

Baseline BUN and SCr values were considered as the blood values obtained 24 h before the operation. Each patient was assigned a pRIFLE class: R (risk of renal dysfunction), I (injury to the kidney), or F (failure of kidney function), according to the postoperative change in creatinine clearance. Classes R, I, and F correspond to a fall in GFR of 25%, 50%, and 75%, respectively, relative to baseline values. Patients with a postoperative GFR of <35 mL/min/1.73 m^2^ were assigned to class F [15].

### Statistical Analysis

Given the exploratory retrospective design and limited sample size, statistical analyses were interpreted cautiously. Because of the limited number of postoperative AKI events, only two variables (cardiopulmonary bypass [CPB] duration and nadir-indexed oxygen delivery [DO_2_]) were retained in the final multivariable logistic regression model to minimize the risk of overfitting. No formal sample size calculation was performed because of the retrospective exploratory nature of the study.

Statistical analyses were performed using SPSS (Statistical Package for the Social Sciences for Windows, version 21.0). Continuous variables are presented as median (interquartile range [IQR]) or mean ± standard deviation, as appropriate, and categorical variables as number (percentage). The normality of data distribution was assessed using the Kolmogorov–Smirnov test. Comparisons between groups were performed using the independent-samples *t*-test or appropriate nonparametric tests for continuous variables and the chi-square or Fisher’s exact test for categorical variables.

Preoperative and intraoperative variables were first evaluated using univariable logistic regression analysis. Variables with a univariable *p* value <0.10 were considered for inclusion in the multivariable logistic regression model. Because CPB duration and aortic cross-clamp duration demonstrated substantial collinearity, only CPB duration was retained in the final model. Receiver operating characteristic (ROC) analysis was performed to identify an exploratory nadir DO_2_ threshold associated with postoperative AKI, and the area under the ROC curve (AUC) was used to assess discriminative performance. The ROC-derived threshold was subsequently evaluated in the multivariable logistic regression model. As a sensitivity analysis, the multivariable model was repeated with DO_2_ entered as a continuous variable rather than dichotomized.

Internal validation was performed using bootstrap resampling with 1000 iterations applied to the final multivariable logistic regression model, which included CPB duration (>120 min) and nadir DO_2_ (<360 mL/min/m^2^). Model discrimination was evaluated by calculating the bootstrap mean area under the ROC curve (AUC) and its 95% bootstrap confidence interval. The reported AUC represents the apparent (mean bootstrap) value and was not optimism-corrected, given the limited sample size; it is therefore presented as an exploratory measure of discriminative stability. All statistical tests were two-sided, and a *p* value <0.05 was considered statistically significant. This study is reported in accordance with the STROBE (Strengthening the Reporting of Observational Studies in Epidemiology) guidelines for observational studies (see Supplementary Materials).

## 3. Results

A total of 40 consecutive patients fulfilled the study eligibility criteria and were included in the final analysis. Complete perioperative and postoperative data required for AKI classification and oxygen delivery calculations were available for all included patients. No patients were excluded after enrollment because of missing outcome data. Sixteen patients (40%) developed AKI according to the pRIFLE criteria (75% risk; 18.7% injury; 6.2% failure; no patients were classified under loss or end-stage renal disease categories).

The preoperative and operative comparison data of patients with and without AKI are shown in Table 1. In patients who developed AKI, the CPB duration (median 110 vs. 90 min) and cross-clamp time (median 65 vs. 50 min) were longer (*p* < 0.05). In patients who developed AKI, the nadir DO_2_ (median 370 vs. 385 mL/min/m^2^) and average DO_2_ (median 390 vs. 400 mL/min/m^2^) were lower (*p* < 0.05).

Postoperative outcomes are presented in Table 2. Compared with the non-AKI group, patients who developed AKI required significantly longer PCICU and hospital stays as well as prolonged mechanical ventilation (all *p* < 0.05). In addition, AKI patients demonstrated higher peak lactate levels, higher maximum 24 h vasoactive inotropic scores, and a greater incidence of low cardiac output syndrome (*p* < 0.05). Importantly, 30-day all-cause mortality was significantly higher in the AKI group than in the non-AKI group (18.7% vs. 4.1%, *p* = 0.02). There were no significant differences between groups regarding postoperative pneumonia, bleeding, hepatic dysfunction, blood glucose levels, or transfusion requirements.

ROC analysis was performed for both nadir and average DO_2_ using AKI as the outcome. For average DO_2_, the AUC was 0.55 with a cut-off of 390 mL/min/m^2^ (sensitivity 60%, specificity 40%). Nadir DO_2_ showed the strongest discrimination, with an AUC of 0.79 (95% CI 0.68–0.87) and a cut-off of 360 mL/min/m^2^ (sensitivity 70%, specificity 80%). In sensitivity analysis, nadir DO_2_ remained independently associated with AKI when entered as a continuous variable (OR 0.36 per 10 mL/min/m^2^ increase, 95% CI 0.18–0.75, *p* = 0.04). The overall model was statistically significant (likelihood ratio test *p* = 0.0003), with a pseudo R^2^ of 0.24.

Bootstrap internal validation of the final multivariable model yielded a mean AUC of 0.82 (95% bootstrap CI 0.68–0.94), indicating stable discriminative performance.

In the univariate analysis, only CPB duration, aortic clamp time, nadir DO_2_, and average DO_2_ during CPB were significant. RACHS-1 category, preoperative cyanosis, pulmonary hypertension, and intraoperative transfusion volume were also evaluated but were not significantly associated with AKI in the univariate analysis. Variables with a univariate *p* value <0.10 were entered into the multivariable analysis. Various factors identified in the univariate analysis were tested for intercorrelation. A significant correlation was found between CPB duration and aortic clamp time, as well as between nadir DO_2_ and average DO_2_. Therefore, CPB duration and nadir DO_2_ were included in the multivariate models because these two variables were independently associated with AKI. After adjusting for other variables, according to the multivariate analysis, long CPB duration (>120 min, Odds Ratio 2.10, 95% CI 1.10–4.00, *p* = 0.032) and nadir DO_2_ (<360 mL/min/m^2^, Odds Ratio: 4.48, 95% Confidence Interval: 1.93–10.40, *p*<0.001) were independently associated with postoperative AKI. The results of multivariable logistic regression analyses are presented in Table 3.

## 4. Discussion

This study explored whether nadir DO_2_ during pediatric CPB is associated with postoperative AKI in our center. We found that prolonged CPB duration and lower nadir DO_2_ values were both related to AKI. Although the link between oxygen delivery and AKI during CPB has been examined in several pediatric studies, the optimal DO_2_ threshold across different ages, surgical groups, and perfusion strategies remains undefined. Our pilot data from a single-center infant cohort add real-world evidence and identify an exploratory nadir DO_2_ threshold broadly in line with previously reported values, supporting the potential value of oxygen delivery monitoring within contemporary perfusion management.

Kidney injury is a recognized complication among children operated on for congenital heart disease with bypass support. When it reaches a severe stage, it tends to accompany longer ventilator dependence, heavier reliance on inotropic agents, and extended stays in both intensive care and the ward. Across studies, reported AKI incidence varies widely from 3% to 42%, and associated mortality has ranged from 20% to 79% [16,17]. Additionally, the incidence may vary depending on the classification system used. In our study, the incidence using the pRIFLE method was found to be 40%. Consistent with the literature, morbidity and mortality were higher in patients who developed AKI compared to those who did not.

Several studies have demonstrated an association between low DO_2_ and postoperative AKI in pediatric CPB, although optimal threshold values remain heterogeneous across different patient populations and perfusion strategies [5,7]. Reviewing 479 children under 15 years of age, Taka et al. [7] documented AKI in 30.7% yet found no meaningful relationship between nadir DO_2_ and AKI, even though lower nadir values coincided with elevated postoperative lactate. Hayward et al. [8] suggested a candidate risk cut-off, tying AKI to nadir DO_2_ below 350 mL/min/m^2^. Drawing on a broader sample of 980 patients, Dreher et al. [9] found that sustaining oxygen delivery above 340 mL/min/m^2^ in neonates and above 400 mL/min/m^2^ in infants accompanied a lower rate of postoperative AKI.

Setting nadir DO_2_ against cystatin C, Vassalos et al. [18] associated readings below 300 mL/min/m^2^ with poorer recovery. Zhang et al. [19], in turn, identified nadir DO_2_ cut-offs of 269 mL/min/m^2^ for arterial switch operations performed under moderate hypothermia and 353 mL/min/m^2^ for those aged between one month and three years as independent markers of adverse outcomes and AKI. In our own cohort, we likewise observed that reduced nadir DO_2_ together with extended CPB duration could be tied to the development of AKI. Because of the limited number of AKI events, the multivariable model was intentionally restricted to two variables to minimize overfitting risk. Moreover, because longer CPB duration may contribute both to reduced oxygen delivery and increased postoperative morbidity, complete separation of these effects is challenging in observational studies. Given the limited number of AKI events, additional clinically relevant variables could not be incorporated into the multivariable model without increasing the risk of overfitting. Therefore, the potential influence of other perioperative risk factors cannot be excluded. In addition, although the RACHS-1 category was not significantly associated with AKI in the present cohort, surgical complexity may still have influenced postoperative outcomes. The absence of a significant association in this study may reflect the limited sample size and consequent lack of statistical power rather than the absence of a true effect.

For adults undergoing CPB, the reported critical DO_2_ ranges from 262 to 300 mL/min/m^2^ [20,21], whereas pediatric data remain scarce [5,15]. Because infants have higher metabolic demands, higher CPB flow rates are generally advised. Bojan et al. [22] observed that the degree of DO_2_ variation tracked with rising lactate and estimated the nadir DO_2_ required to sustain aerobic metabolism in neonates during normothermic CPB at 340 mL/min/m^2^. Similarly, Zhang et al. [5] reported a risk of AKI when nadir DO_2_ dropped below 353 mL/min/m^2^. In our cohort, ROC analysis identified 360 mL/min/m^2^ as an exploratory nadir DO_2_ threshold associated with postoperative AKI. Because this threshold was both derived and evaluated within the same retrospective cohort, its apparent diagnostic performance should be interpreted with caution. Because internally derived thresholds are subject to optimistic bias, the reported sensitivity, specificity, and odds ratio may overestimate the true predictive performance. This finding should therefore be considered a preliminary, hypothesis-generating observation with no implication of clinical applicability and requires validation in larger independent prospective cohorts.

Interest in goal-directed perfusion has grown steadily, an approach that relies on real-time tracking of oxygen-metabolism markers—among them, DO_2_ and the oxygen extraction index—while simultaneously titrating hemodilution and pump output to steer the conduct of bypass [23]. GDP facilitates early recognition of hypoperfusion and anaerobic metabolism, allowing timely intervention to preserve tissue perfusion, an approach we also follow in our unit. Clinically, continuous oxygen delivery monitoring may offer an additional means of identifying periods of inadequate perfusion and supporting intraoperative decisions. Future prospective multicenter studies should determine whether maintaining DO_2_ above validated thresholds reduces postoperative AKI and improves outcomes in pediatric cardiac surgery.

### Limitations

A number of constraints apply to this work. To begin with, it was carried out retrospectively at a single institution in a modest number of infants operated on for congenital heart disease, which may restrict how far the results extend to other patient groups and care environments. Second, despite the inclusion of relevant perioperative variables, residual confounding from unmeasured factors, including variations in perioperative hemodynamic management, arterial pressure targets, and vasoactive medication use, cannot be excluded. Third, the heterogeneous spectrum of congenital heart defects and surgical procedures may have influenced postoperative renal outcomes. In addition, most AKI cases were classified within the mildest pRIFLE “Risk” category. The small number of severe AKI events precluded meaningful subgroup analyses and prevented separate evaluation of clinically significant AKI. Accordingly, the generalizability of these findings to more severe forms of AKI may be limited. Furthermore, core temperature monitoring was performed using different anatomical sites (nasopharyngeal, bladder, or rectal), which may have introduced measurement variability during hypothermic cardiopulmonary bypass. Finally, although internal validation was performed using bootstrap resampling, this was not optimism-corrected, and the ROC-derived DO_2_ threshold should still be interpreted cautiously because of the limited sample size, the exploratory nature of the analysis, and the absence of external validation. Because the threshold was both derived and evaluated within the same cohort, internally derived thresholds of this kind remain subject to optimistic bias, and the reported diagnostic performance may overestimate the true values and may not be reproducible in independent populations. Consistent with STROBE recommendations, these findings should be regarded as hypothesis-generating rather than confirmatory and warrant validation in larger prospective multicenter studies.

## 5. Conclusions

In this pilot cohort of infants undergoing congenital heart surgery, lower nadir DO_2_ values during cardiopulmonary bypass were associated with postoperative AKI. An exploratory ROC-derived threshold of 360 mL/min/m^2^ was identified; however, because this threshold was derived and evaluated within the same cohort, its diagnostic performance should be interpreted with caution. At present, this threshold represents a hypothesis-generating observation only, without any implication of clinical applicability, and requires validation in larger prospective, independent cohorts. Further studies with larger sample sizes are needed to better understand the relationship between DO_2_ and AKI in this population.

## Figures and Tables

**Table 1:** Preoperative and Operative Comparison Data of Patients with and without Acute Kidney Injury (AKI).

Variables	Non-AKI (*n* = 24)	AKI (*n* = 16)	*p* Value
**Patients, %**	60	40	NS
**Age, month**	5 (4–6)	7 (4–9)	NS
**Male**	12 (50)	8 (50)	NS
**Weight, kg**	5.5 (4.5–6.5)	6.5 (5.6–8)	NS
**Body Surface area, m^2^**	0.43 ± 0.14	0.60 ± 0.10	NS
**RACHS-1 ≥ 4**	12 (50)	10 (63)	NS
**Preoperative Cyanosis**	8 (33)	5 (31.2)	NS
**Preoperative Pulmonary Hypertension**	13 (54.1)	9 (56.2)	NS
**Serum Creatinine (mg/dL)**	0.4 (0.3–0.5)	0.42 (0.35–0.48)	NS
**Hemoglobin, g/dL**	11.5(10–12.5)	12(10–14)	NS
**CPB time, minutes**	90 (75–110)	110 (100–120)	**0.001**
**Cross-clamp time, minutes**	50 (40–60)	65 (55–75)	**0.003**
**Volume of packed red blood cells transfused** **during surgery, mL**	300 (250–350)	300 (250–350)	NS
**Nadir DO_2_ on CPB, mL/min/m^2^**	385 (380–400)	370 (360–380)	**<0.001**
**Average DO_2_ on CPB, mL/min/m^2^**	400(390–410)	390 (380–400)	**0.008**
**Lowest hemoglobin on CPB, g/dL**	8 (7–9)	7.5 (7–8)	NS
**Lowest temperature on CPB, °C**	33.7 (33.1–34.2)	33.5 (33.1–33.8)	NS
**Mean arterial pressure on CPB, mmHg**	45 (40–55)	47 (42–52)	NS
**Peak blood lactate, mmol/L**	2.8 (2.4–3.5)	3 (2.7–3.6)	NS
**Peak blood glucose, mmol/L**	8.5 (7.5–9.5)	9 (8–10)	NS

*n* (%) or Median (IQR); CPB: Cardiopulmonary Bypass; NS: not significant.

**Table 2:** Postoperative comparison data of cases with and without acute kidney injury.

Variables	Non-AKI	AKI	*p* Value
**PCICU stay, day**	3 (2–6)	7 (3–10)	**0.011**
**Hospital stay, day**	9 (7–13)	15 (9–22)	**0.002**
**MV time, day**	0 (0–1)	1 (0–6)	**0.005**
**Volume of packed red blood cells transfused in PCICU, mL**	30 (0–120)	50 (0–240)	NS
**24 h maximum VIS**	0 (0–5)	6 (0–10)	**0.002**
**Peak blood lactate in PCICU, mmol/L**	2.1 (1.6–2.6)	2.8 (1.9–3.2)	**0.020**
**Peak blood glucose in PICU, mmol/L**	7.1 (6.3–8.2)	7.6 (6.8–9.3)	NS
**LCOS**	4 (16.6)	7 (43.7)	**0.010**
**Pneumonia**	4 (16.6)	3 (18.7)	NS
**Bleeding**	2 (8.3)	2 (12.5)	NS
**Hepatic dysfunction**	0 (0)	1(6.2)	NS
**All-cause mortality within 30 days after surgery**	1 (4.1)	3 (18.7)	**0.02**

*n* (%) or Median (IQR); LCOS: Low Cardiac Output Syndrome; MV: Mechanical ventilation; NS: not significant; PCICU: Pediatric Cardiac Intensive Care Unit; VIS: vasoactive inotropic score.

**Table 3:** Multivariable Logistic Regression Analysis for Postoperative AKI.

Variables	Odds Ratio (OR)	95% Confidence Interval (CI)	*p* Value
**CPB duration >120 min**	2.10	1.10–4.00	**0.032**
**Nadir DO_2_ <360 mL/min/m^2^**	4.48	1.93–10.40	**<0.001**

CPB: cardiopulmonary bypass; DO_2_: oxygen delivery.

## Data Availability

The data presented in this study are available on request from the corresponding author. The data are not publicly available due to privacy and ethical restrictions.

## Supplementary Materials

The following supporting information can be downloaded at: https://www.mdpi.com/article/10.3390/children13070893/s1, STROBE Statement—checklist of items that should be included in reports of observational studies.
