# Supplementary material for: Nadir Oxygen Delivery During Pediatric Cardiopulmonary Bypass and Postoperative Acute Kidney Injury: A Pilot Cohort Study
Source: Children (Basel). 2026 Jul 3;13(7):893. doi: 10.3390/children13070893 (PMC13406364; doi:10.3390/children13070893)
Supplement: Supplementary file 1 [file children-13-00893-s001.zip › children-4379830-supplementary.pdf]

STROBE Statement—checklist of items that should be included in reports of observational studies

	Item No.	Recommendation	Page No.	Relevant text from manuscript
**Title and abstract**	1	(*a*) Indicate the study's design with a commonly used term in the title or the abstract	1	Title identifies the study as a retrospective observational cohort study
		(*b*) Provide in the abstract an informative and balanced summary of what was done and what was found	1	Structured abstract including background, methods, results and conclusions
**Introduction**				
Background/rationale	2	Explain the scientific background and rationale for the investigation being reported	2	Background and rationale for evaluating DO_2 and AKI during pediatric CPB
Objectives	3	State specific objectives, including any prespecified hypotheses	2	Study objective stated in final paragraph of Introduction
**Methods**				
Study design	4	Present key elements of study design early in the paper	3	"This retrospective study included pediatric patients..."
Setting	5	Describe the setting, locations, and relevant dates, including periods of recruitment, exposure, follow-up, and data collection	3	Study setting, dates (October–December 2024), institution and data collection described
Participants	6	(*a*) *Cohort study*—Give the eligibility criteria, and the sources and methods of selection of participants. Describe methods of follow-up<br>*Case-control study*—Give the eligibility criteria, and the sources and methods of case ascertainment and control selection. Give the rationale for the choice of cases and controls<br>*Cross-sectional study*—Give the eligibility criteria, and the sources and methods of selection of participants	3	Inclusion and exclusion criteria; participant selection methods described
		(*b*) *Cohort study*—For matched studies, give matching criteria and number of exposed and unexposed<br>*Case-control study*—For matched studies, give matching criteria and the number of controls per	N/A	

		case		
Variables	7	Clearly define all outcomes, exposures, predictors, potential confounders, and effect modifiers. Give diagnostic criteria, if applicable	3-5	Definitions of AKI, DO_2, RACHS-1, VIS and study variables
Data sources/ measurement	8*	For each variable of interest, give sources of data and details of methods of assessment (measurement). Describe comparability of assessment methods if there is more than one group	4-5	Measurement methods, blood gas analysis, perfusion variables and DO_2 calculation
Bias	9	Describe any efforts to address potential sources of bias	5-6	Consecutive eligible patients were included using predefined inclusion and exclusion criteria. Standardized definitions (pRIFLE criteria) were used for AKI assessment. To reduce model instability and overfitting, the multivariable model was restricted to two variables and collinearity between predictors was evaluated.
Study size	10	Explain how the study size was arrived at	5	Retrospective pilot study; no formal sample size calculation performed

Continued on next page

Quantitative variables	11	Explain how quantitative variables were handled in the analyses. If applicable, describe which groupings were chosen and why	5-6	Handling of quantitative variables and ROC-derived cutoff analysis
Statistical methods	12	(*a*) Describe all statistical methods, including those used to control for confounding	5-6	Statistical methods and multivariable logistic regression described
		(*b*) Describe any methods used to examine subgroups and interactions	N/A	
		(*c*) Explain how missing data were addressed	4–5	Missing data handling described
		(*d*) *Cohort study*—If applicable, explain how loss to follow-up was addressed<br>*Case-control study*—If applicable, explain how matching of cases and controls was addressed<br>*Cross-sectional study*—If applicable, describe analytical methods taking account of sampling strategy	3, 6	No patients lost to follow-up; complete outcome data available
		(*e*) Describe any sensitivity analyses	N/A	
**Results**				
Participants	13*	(a) Report numbers of individuals at each stage of study—eg numbers potentially eligible, examined for eligibility, confirmed eligible, included in the study, completing follow-up, and analysed	6	Total number of eligible patients included in the final analysis and AKI events reported.
		(b) Give reasons for non-participation at each stage	3, 6	Inclusion and exclusion criteria, along with reasons for non-participation, are described.
		(c) Consider use of a flow diagram	N/A	
Descriptive data	14*	(a) Give characteristics of study participants (eg demographic, clinical, social) and information on exposures and potential confounders	6-8	Baseline demographic, clinical and operative characteristics presented in Tables 1 and 2
		(b) Indicate number of participants with missing data for each variable of interest	4-5	Missing data reported for two isolated perfusion measurements
		(c) *Cohort study*—Summarise follow-up time (eg, average and total amount)	N/A	
Outcome data	15*	*Cohort study*—Report numbers of outcome events or summary measures over time	6-8	AKI incidence and postoperative outcomes reported
		Case-control study—Report numbers in each exposure category, or summary measures of exposure		
		Cross-sectional study—Report numbers of outcome events or summary measures		
Main results	16	(*a*) Give unadjusted estimates and, if applicable, confounder-adjusted estimates and their precision	8-9	Univariate and multivariable

	(eg, 95% confidence interval). Make clear which confounders were adjusted for and why they were included		logistic regression results with ORs and 95% CIs
	(*b*) Report category boundaries when continuous variables were categorized	7-9	ROC-derived cutoff values and categorized variables reported
	(*c*) If relevant, consider translating estimates of relative risk into absolute risk for a meaningful time period	N/A	

Continued on next page

Other analyses	17	Report other analyses done—eg analyses of subgroups and interactions, and sensitivity analyses	N/A	No subgroup, interaction, or sensitivity analyses were performed.
**Discussion**				
Key results	18	Summarise key results with reference to study objectives	9-10	Key findings summarized in Discussion
Limitations	19	Discuss limitations of the study, taking into account sources of potential bias or imprecision. Discuss both direction and magnitude of any potential bias	10-11	Limitations section
Interpretation	20	Give a cautious overall interpretation of results considering objectives, limitations, multiplicity of analyses, results from similar studies, and other relevant evidence	9-11	Interpretation of findings in context of previous literature
Generalisability	21	Discuss the generalisability (external validity) of the study results	10-11	Generalizability discussed in Limitations section
**Other information**				
Funding	22	Give the source of funding and the role of the funders for the present study and, if applicable, for the original study on which the present article is based	12	This research received no external funding

*Give information separately for cases and controls in case-control studies and, if applicable, for exposed and unexposed groups in cohort and cross-sectional studies.
